# Stem cell therapy for hepatocellular carcinoma and end-stage liver disease

**DOI:** 10.1186/s43046-023-00194-z

**Published:** 2023-11-06

**Authors:** Mona S. Abdellateif, Abdel-Rahman N. Zekri

**Affiliations:** 1 Medical Biochemistry and Molecular Biology, Cancer Biology Department, National Cancer Institute, Cairo University, Cairo, 11976 Egypt; 2 Molecular Virology and Immunology Unit, Cancer Biology Department, NCI, Cairo University, Cairo, 11976 Egypt

**Keywords:** HCC, Liver, MSCs, Stem cell and end stage

## Abstract

Hepatocellular carcinoma (HCC) is a major health problem worldwide, especially for patients who are suffering from end-stage liver disease (ESLD). The ESLD is considered a great challenge for clinicians due to the limited chance for liver transplantation, which is the only curative treatment for those patients. Stem cell-based therapy as a part of regenerative medicine represents a promising application for ESLD patients. Many clinical trials were performed to assess the utility of bone marrow-derived stem cells as a potential therapy for patients with liver diseases. The aim of the present study is to present and review the various types of stem cell-based therapy, including the mesenchymal stem cells (MSCs), BM-derived mononuclear cells (BM-MNCs), CD34 + hematopoietic stem cells (HSCs), induced pluripotent stem cells (iPSCs), and cancer stem cells.

Though this type of therapy achieved promising results for the treatment of ESLD, however still there is a confounding data regarding its clinical application. A large body of evidence is highly required to evaluate the stem cell-based therapy after long-term follow-up, with respect to the incidence of toxicity, immunogenicity, and tumorigenesis that developed in many patients.

## Introduction

Hepatocellular carcinoma (HCC) is the most common cause of cancer-related death all over the world. It ranked third for mortality and fifth in incidence according to the World Health Organization’s (WHO) statistics in 2020 [1]. The incidence of HCC achieved about a 75% rise in the last two decades, and it is continuously increasing, where males are three times more likely to be affected than females [2]. It is expected that more than one million deaths due to liver cancer will occur by 2030 [3].

There are many risk factors for HCC that differ between developed and developing countries. E.g., chronic hepatitis B virus (HBV) infection and aflatoxin B1 (AFB1) are the major risk factors for liver cancer in developing regions [4, 5], while hepatitis C virus (HCV) [6] and nonalcoholic fatty liver disease (NAFLD) are the major risk factors for liver cancer in developed countries [7].

The outcome of HCC patients is usually poor, as surgery is suitable only for early-stage patients who represent 5–15% of the patients, in which the risk of postoperative complications is more common due to diminished hepatic regenerative capacity, whereas the treatment strategy for patients with intermediate-stage liver cancer is mainly trans-arterial chemoembolization (TACE), which achieves only a 23% improvement in the 2-year survival rate [8].

In the last 15 years, there are numerous molecular-targeted drugs that have been approved by the FDA for the treatment of patients with advanced HCC. These drugs included kinase inhibitors such as sorafenib (2008), regorafenib (2017), lenvatinib (2018), and cabozantinib (2019). Also, angiogenesis inhibitors such as ramucirumab (2019) and bevacizumab (2020), as well as immune checkpoint inhibitors including pembrolizumab (2018), atezolizumab (2020), and nivolumab (2020) [9]. However, still the incidence of recurrence and mortality rates are steadily increasing, where the efficacy of these drugs is modest and can extend the survival rates for only a few months in advanced HCC patients [10]. In addition to the emergence of drug toxicity or inefficacy that emerged after long-term use [11].

Liver transplantation is an ideal option of treatment for selected patients with HCC; however, lacking donors, high cost, and prolonged administration of immunosuppressive drugs make it of limited use [12, 13]. As a result, still there is no effective line of therapy that could improve the prognosis and the outcome of patients suffering from HCC [11]. However, the emerging stem cell therapy could open a new avenue for HCC patients especially those with end-stage liver disease (ESLD). Many types of research are developing now to maintain the optimum conditions for producing an effective and potent stem cell therapy for HCC patients. Hence, the aim of the current study was to review the various types of stem cell-based therapy including mesenchymal stem cells (MSCs), cancer stem cells (CSCs), autologous bone marrow-derived cells, and induced pluripotent stem cells (iPSCs).

## Stem cells definition and classification

Stem cells are unspecialized cells that can differentiate into different types of cells, in addition to their ability of self-renewal in order to maintain stem cell populations in different tissues [14]. Stem cells can be classified according to the differentiation potential into totipotent, pluripotent, multipotent, and unipotent cells [15]. The most potent one which has the highest differentiation potential is the totipotent stem cell. These totipotent stem cells are the early blastomeres that are formed 1–3 days after oocyte fertilization, and they can form both embryo and extraembryonic structures [16]. The next type is the pluripotent stem cells (PSCs), which can differentiate into all germ layers. It is formed of the embryonic stem cells (ESCs) which constitute the inner cell mass of the blastocyst (formed 4–14 days after fertilization). These PSCs are capable of the formation of ectoderm, mesoderm, and endoderm, but not extraembryonic structures [17]. After that, these cells are converted to multipotent stem cells, which can differentiate into only all cell types of one germ lineage, while the unipotent stem cells can differentiate only into one cell type [16, 18].

Another classification of stem cells depends upon the origin of the cells; this classification includes (1) embryonic stem cells (ESCs), which are derived from the inner cell mass of the blastocyst, and (2) adult stem cells, which are present in the whole body after development. The latter are multipotent stem cells that function to maintain healing, growth, and replacement of any dead or lost cells [14, 19, 20]. An important type of adult stem cells is the mesenchymal stem cells (MSCs), which act for replenishment and renewing of the tissues in which they reside. They are present mainly in the bone marrow, adipose tissue, hair follicle, and dental pulp [21]. Signals that are controlling stem cell specialization can be divided into external signals, such as physical contact between cells or chemical secretion of certain chemokines by the surrounding tissue, whereas the internal signals are regulated through specific genes in the MSCs [14]. Though MSCs have a limited capacity for differentiation, recent evidence has shown the possibility of restoring the pluripotent differentiation capacity in adult stem cells by forcing the expression of four transcription factors (TFs) that characterize a pluripotent cell [22]. These TFs allow reprogramming of the MSCs and therefore the formation of induced pluripotent stem cells (iPSCs) that can differentiate into the three embryonic layers [15, 23]. The iPSCs have promising applications in regenerative medicine, as it has been now successfully recruited for the treatment of stroke [24], macular degeneration [25], osteoarthritis [26], diabetes, and neurodegenerative diseases [27]. Additionally, it has been investigated for the treatment of many types of cancers including glioma, breast, and hepatic cancer [28–30].

## Cancer stem cells (CSCs) in hepatocellular carcinoma

HCC is considered a complex disease formed of heterogenous cell populations that vary in their molecular, biological, and immunological characteristics. Consequently, this heterogeneity could have a potential effect on the disease recurrence, resistance to treatment, and the clinical outcome of the patients [9, 31]. An accumulated body of evidence suggested that the heterogeneity within HCC is due to a subpopulation of progenitor cells called CSCs. These cells have the capability of self-renewal and plasticity, which allow it to differentiate into different types of cells. Accordingly, these unique features render these liver CSCs (LCSCs) to be responsible for the tumorigenesis, angiogenesis, and metastasis that eventually lead to tumor recurrence and drug resistance [32, 33]. Moreover, Zheng and his colleagues performed the combined transcriptomic and functional analysis at a single-cell level in HCC patients. They found a diversity of LCSCs subpopulations that varies in their molecular, functional, and phenotypic characteristics, which is responsible for the intertumoral heterogeneity that occurs in HCC [34]. Hence, it is important to target these LCSCs in order to improve patients’ response to treatment and survival outcomes [35, 36].

### Characterization of the LCSCs

Extensive research was performed to investigate the identification and characterization of the LCSCs by fluorescence or magnetic-activated cell sorting, through the expression of many surface markers including CD133, CD44, CD90, CD24, CD34, CD47, C-kit, cytokeratin 19 (CK19), epithelial cell adhesion molecule (EpCAM), and intercellular adhesion molecule-1 (ICAM-1) [9, 33, 37–40]. It was demonstrated by Yang et al. that increased serum levels of LCSCs markers including cytokeratin 19 (CK19), KRABCG2, CD133, nestin, and CD44 associated significantly with angiogenesis and inferior outcome of HCC patients [41]. Additionally, it was found that CK19, c-kit, ABCG2, and ALDH have an important role in maintaining tumorigenesis and resistance to radiotherapy or chemotherapy by regulating the expression of drug-efflux-related genes [42–44].

### Circulating CSCs

Another entity concerning CSCs research is the study of circulating CSCs in liquid biopsy for assessing the diagnosis, prognosis, and survival rates of the patients. In this regard, it was observed that increased plasma levels of CD45^−^ ICAM1^+^ LCSCs in HCC patients associated significantly with poor clinical outcomes [45]. Additionally, the plasma level of circulating EpCAM + LCSCs was found to be a useful predictor biomarker for postoperative HCC relapse [46, 47]. Moreover, Guo et al. reported a panel of LCSCs markers formed of EpCAM, CD90, CD133, and CK19 that could efficiently have a role in the early diagnosis and early recurrence of HCC after resection [48].

### Targeting the LCSCs in clinical practice

In the past few years, research has been directed towards targeting the LCSCs through developing anti-surface marker antibodies, oncolytic viruses, epigenetic regulators, and small molecule inhibitors that could selectively affect the LCSCs [49]. The small-molecule inhibitors were directed against certain signaling pathways that regulate the stemness and proliferation of the LCSCs such as Wnt/β-catenin (OMP-18R5 and OMP-54F28) [50, 51], Notch pathway (PF-03084014) [52], TGF-β pathway (LY2157299) [53], and Hedgehog signaling pathway (LED225 [ClinicalTrials.gov. NCT02151864)]), while the anti-surface markers included targeting the CD133 through oncolytic measles viruses (MV-141.7 and MV-AC133) [54] and anti-EpCAM (VB4-845) [55]. Other studies assessed the role of epigenetic control inhibitors on the tumorigenesis and aggressiveness of LCSCs such as zebularine (DNMT1 inhibitor) [56] and SBHA (HDAC inhibitor) [57]. Other clinical trials (ClinicalTrials.gov. NCT02279719) were also conducted including the combination of napabucasin (a STAT3 inhibitor) and sorafenib, or amcasertib (a NANOG inhibitor) and sorafenib, where NANOG is a transcriptional factor that maintains embryonic stem cells pluripotency [9]. Though all the previously mentioned studies achieve a primary suppression of HCC, especially when combined with chemotherapeutic agents, however, all the targeted markers and molecular pathways in LCSCs are similar to the other normal stem cell populations. Hence, eradication of LCSCs may also affect the normal hepatic stem cells which would result in the reduction of hepatic regeneration capacity and consequently liver failure. Therefore, proper identification and specification of the LCSCs remain a challenging matter, and further research is highly required to accurately identify and target the LCSCs [9, 48].

### LCSCs and immunotherapy

Accumulating evidence suggested that the aggressiveness of the LCSCs is due to their poor immunogenicity which allows them to evade immunosurveillance through their interaction with the tumor microenvironment and the inhibition of different immune cells [9]. Therefore, many recent studies tried to assess the utility of these cells in immunotherapy including the development of dendritic cell (DC) vaccine pulsing with CD133 (ClinicalTrials.gov. NCT02049489). In this trial, patients showed an efficient cytotoxic T-cell response against CD133^+^ LCSCs that inhibited tumor growth [58]. Another study was performed by Choi et al. who induced a potent immune cytotoxic T-cell response against CD44^+^ EpCAM^+^ LCSCs using DCs pulsed with CD44 and EpCAM peptides [59]. Other immunotherapeutic modalities which currently under trials are the engineered chimeric antigen receptor (CAR) T cells. One of these studies was the development of CD133-directed CAR T cells in the treatment of patients with advanced HCC [60]. Though these studies provided a good clinical response in controlling the tumor growth and achieving complete remission, the encountered cytotoxicity including the decreased levels of hemoglobin, platelets, and lymphocytes still needs to be resolved [60].

## Clinical applications of MSCs in liver diseases

### The allogenic MSCs

The MSCs are pluripotent non-hematopoietic stem cells that can be isolated from several sources including liver, umbilical cord, placenta, muscle, skin, synovial membrane, amniotic fluid, and tooth root [61, 62]. The MSCs commonly express surface markers including CD73, CD105, and CD90, while they are lacking the expression of CD45, CD34, CD14 or CD11b, CD79α or CD19, and HLA-DR [63]. Various experimental studies and clinical trials had been conducted to investigate the therapeutic utility of MSCs in different diseases including multiple sclerosis (MS), corneal disease, myocardial infarction, Crohn’s disease, amyotrophic lateral sclerosis, and acute respiratory distress syndrome (ARDS) [64–66]. Also, it had been approved in many counties for preclinical and clinical purposes for the treatment of, e.g., graft-versus-host disease (GVHD) in the USA [67] and for the treatment of traumatic or degenerative osteoarthritis in Korea [68].

Some studies assessed the intravenous injection of umbilical cord-derived MSCs (UC-MSCs) in patients with primary biliary cirrhosis (PBC). They reported that the UC-MSCs treatment is a safe and efficient therapy, as there were reduced serum levels of alkaline phosphatase (ALP) and gamma-glutamyl transferase (GGT). However, there were no significant changes in serum glutamic pyruvic transaminase (SGPT), serum glutamic oxaloacetic transaminase (SGOT), albumin, prothrombin time activity, and immunoglobulin M levels [69, 70]. Other clinical trials demonstrated that intravenous infusion of UC-MSCs in patients with liver cirrhosis and HBV injured liver was safe, tolerable, and increased the survival rates of the patients [71–74]. On the other hand, Nevens et al. conducted an open-label phase II clinical study (EudraCT 2016–001177-32) on 24 patients with acute-on-chronic liver failure (ACLF) treated with human MSCs (HepaStem) transplantation. They reported improved survival with no adverse events related to therapy [75]. Similarly, Lin et al. performed an open-label non-blinded randomized controlled study on 110 patients with HBV-related ACLF infused with 1.0–10 × 10^5^ cells/kg allogeneic bone marrow-derived mesenchymal stem cells (BM-MSCs) and followed up for 6 months. Patients treated with the BM-MSCs showed reduced mortality rates due to decreased incidence of infection and improved liver function compared to the control group [76]. These data provide evidence that the BM-MSCs could be a potential safe and feasible therapeutic option for HBV-related ACLF patients.

Though exogenous stem cell therapy is considered by different companies all over the world for sale, its safety and efficacy are still major challenges in large-scale clinical trials that lead to the inapplicability of allogenic MSCs [77]. These challenges included mainly the immunogenic incompatibility that developed either early or secondary after repeated infusions due to the accumulation of inflammatory cells and mediators such as interferon-γ [78, 79]. Other technical problems associated with the allogenic MSCs are poor-quality control and lack of stability, in addition to the inconsistent heterogeneity, differentiation, and migratory capacity of the cells [80, 81].

### The autologous MSCs therapy

To date, cellular therapy including MSCs has become a promising therapeutic strategy for patients with decompensated liver disease [82]. In fact, the MSCs have many advantages that make them a unique line of treatment for those patients with ESLD. As these cells were obtained from the patient himself, therefore, all the differentiated cells will carry the same genetic profile of the patient. In addition, MSCs are characterized by low immunogenicity because they express low levels of major histocompatibility complex-1 (*MHC-I*) molecules, so they overcome the immune rejection occurred with liver transplantation [12, 83].

Furthermore, the delivered MSCs exert an inhibitory effect on HCC through different mechanisms including restoration of functioning hepatocytes, antifibrotic, antiapoptotic, and antioxidative effects. Additionally, it was found that these effects were potentiated through co-treatment of the MSCs with melatonin [84, 85]. Also, they exert anti-inflammatory function through increasing the secretion of interleukin-10 (*IL-10*), indoleamine 2,3‐dioxygenase (*IDO*), prostaglandin 2 (*PGE2*), transforming growth factor (*TGF)‐β3*, and hepatocyte growth factor (*HGF*) [86]. Moreover, the MSCs have an antitumor effect via inhibition of the Wnt signaling pathway [87]. Another important mechanism is the immunomodulation characteristic of MSCs as they suppress the immune response through inhibiting T-cell activation and proliferation, as well as inducing macrophages shift from M1 to M2 [88, 89]. Also, the increased levels of anti-inflammatory mediators lead to suppressing the effector T cells and stimulating the regulatory T cells through increasing *FOXP3*, *CTLA4*, and *GITR* expression [90, 91]. Therefore, Zhang and his colleagues performed a clinical trial (no. ChiCTR2000037732), in which they injected six doses of MSCs 1 × 10^6^/kg bodyweight intravenously for patients with ABO-incompatible liver transplantation (ABO-i LT). They found that MSC transfusion could efficiently reduce the risk of acute rejection similar to that of rituximab treatment. Additionally, MSCs are preferred as an immunosuppressive approach for ABO-i LT because there is no risk of infection and biliary complications that might be associated with rituximab [92].

Another concern regarding MSCs therapy is the ability of these cells to move in the direction of the inflammatory or damaged area to make tumor homing [25]. This attraction is maintained through the increased production of certain factors by the tumor cells including IL-6, platelet-derived growth factor subunit B (PDGFB), vascular endothelial growth factor (VEGF), and transforming growth factor beta-1 (TGF-b1) [93, 94]. Recently, it was reported that some chemokines have a major role in MSCs tumor homing including C-X-C motif chemokine receptor 4 (CXCR4), CCR1, CXCR5, CXCR6, CCR7, and CCR9 [95–97]. These migratory and homing properties of MSCs allow them to be a promising vehicle for the delivery of anticancer molecules. This strategy was applied through either loading the MSCs with drug molecules or nanoparticle carriers. In this regard, Zhao et al. assessed the efficacy of adipose-derived MSCs (AD-MSCs) loaded with superparamagnetic iron oxide-coated gold nanoparticles (*SPIO@AuNPs*) in HCC cell line and in mice with induced liver injury. They reported a successful homing of *SPIO@AuNP*-loaded AD-MSCs in the hepatic tissue, which make the AD-MSCs a potential specific delivery of therapeutic agents in patients with liver diseases [98]. Another approach was developed through genetic modification of MSCs to stimulate the expression of tumor suppressor genes or anticancer proteins [99]. Schug and his team demonstrated that genetically engineered MSCs could significantly decrease tumor proliferation and increase survival rate in mice treated with *SMAD-NIS-MSCs* through *TGFB1*-induced *SMAD* promoter activity [100]. However, these techniques are studied experimentally in vivo and still not recruited for clinical applications in liver cancer patients.

In the past few years, many clinical trials have been performed to assess the efficiency of autologous MSCs in the treatment of liver diseases [101–104]. Among these trials was that done by Suk et al., who reported that BM-MSCs therapy could improve liver function and Child–Pugh score in patients with liver cirrhosis compared to the control group. They did not find any adverse event associated with the BM-MSCs administration for 12-month follow-up period [102]. Another clinical trial was performed by Sakai and his team (ClinicalTrials.gov. NCT01062750), where they used autologous adipose tissue-derived stem cells (ADRCs) for intrahepatic arterial infusion in patients with liver cirrhosis. The results showed that ADRCs therapy could efficiently and safely repair liver cirrhosis [103].

Though the autologous MSCs-based therapy provides a promising strategy for regenerative treatment of liver disease, there are many limitations encountered. These limitations are the emerged chromosomal instability, emboli formation, and inducing immune reaction, in addition to the incidence of unwanted differentiation and tumor formation [105, 106]. Other reported limitations were the low migration and poor survival of the transplanted MSCs, which directed researchers to consider other stem cells for the treatment of liver diseases [107, 108].

## Autologous bone marrow-derived cells

Bone marrow (BM)-derived CD34 + hemopoietic stem cells (HSCs) or whole mononuclear cells (BM-MNCs) were considered an attractive therapeutic approach for ESLD patients [109]. It had been reported that transplantation of autologous BM-MNCs is a safe and feasible option for patients with decompensated alcoholic liver diseases. Additionally, the end-stage liver disease (MELD) scores and liver function were improved; however, there was an insufficient regenerative capacity [110, 111]. These results are comparable to that reported by Lyra et al., who concluded that infusion of BM-MNCs via hepatic artery could significantly increase albumin level and improve the Child–Pugh score, while there was no change in the MELD score [112]. Another study is done by Mohamadnejad et al. (ClinicalTrials.gov. NCT01120925), who compared intraportal infusion of CD133 + cells, BM-MNCs, and placebo group. They found that there was a transient improvement in the MELD score in patients receiving CD133 + cells after a follow-up period of 3 months, while there was no improvement in the MELD score after 6 months of follow-up period. Also, they concluded that there was no significant improvement in the MELD score of patients infused with MNCs neither after 3 nor 6 months of follow-up [113].

Indeed, most of the performed clinical trials reported a benefit of autologous BM stem cell transplantation after a maximum of 1 year [83]. However, in a recent study done by Kim et al. [114], who followed up patients for five years after autologous BM stem cell transplantation. They observed the development of malignant tumors in 36.8% (7/19) of the patients, in the form of HCC in 26.3% (5/19), lymphoma in 5.3% (1/19), and colon cancer in 5.3% (1/19) of the patients. Zekri et al. reported that intraportal infusion with CD34 + CD133 + cells, followed by peripheral IV infusion of in vitro-differentiated MSCs within 1 week, and repeated infusion after 3 months achieved a beneficial therapeutic effect on the patients, with minimal adverse events and prolonged clinical efficacy [115]. Similarly, many other studies reported that G-CSF-mobilized CD133^+^ stem-progenitor cells (SPCs) could induce transient improvement in ESLD patients with no detectable adverse events [116–118].

On the other side, Chruscinski and his colleagues observed high mortality and morbidity rates in patients with HSTs liver transplantation after follow-up for a long period. They concluded that HSTs could not be considered for clinical applications at this time due to increased incidence of multiorgan failure and toxicity after discontinuation of the immunosuppressive regimen [119]. Similarly, Margini et al. concluded that the therapeutic effect of HSCs is only temporary, which suggests that HSCs act through producing trophic support rather than trans-differentiation [116].

## Induced pluripotent stem cell (iPSC)

Induced pluripotent stem cells (iPSCs) are produced from adult somatic cells (usually fibroblast) that have been genetically reprogrammed to differentiate into pluripotent ESC [120]. This reprogramming occurs by transfection with four transcription factors called Yamanaka factors (OSKM; Oct4, Sox2, Klf4, and c-Myc) [108]. These iPSCs were developed to overcome the challenges accompanying the application of other types of stem cells. As they provide a reproducible and reliable source of expandable, bankable, and engraftable hepatocyte-like cells (HLCs), which can be repeatedly used for clinical treatments [121]. Additionally, Antarianto et al. reported that using HLCs differentiated from iPSCs in vitro is more mature with lower cell–ECM adhesion, spatial cell distribution, albumin secretion, and CYP450 expression than HLCs that differentiated from MSCs in decellularized liver scaffold [122].

Many experimental studies reported the functionality and feasibility of iPSCs in different liver diseases including acute liver failure and liver fibrosis [123, 124]. Moreover, advanced therapeutic technology was developed in the field of gene editing modality that utilized the iPSCs for treating patients with metabolic liver diseases. These genetically engineered iPSCs showed promising success in disease modeling and gene correction in different hereditary liver diseases including Crigler-Najjar disease or alpha-1 antitrypsin (A1AT) deficiency, Wilson’s disease, familial hypercholesterolemia, glycogen storage disease type 1, Niemann-Pick type C, and hemophilia B [125–132]. Though there are many clinical trials in phases I or II being conducted on several diseases including cardiovascular and neurological disorders [133, 134], however, no trials concerned with liver diseases were registered until now [135]. Indeed, there are many critical aspects which should be considered before transferring these cells safely for clinical applications including the long-term stability and tolerability. In addition to the increased risk of immunological reaction and tumorigenesis especially in those with genetic modification [121, 136].

## Conclusion

In conclusion, stem cell-based therapy achieved promising results regarding improving liver function, MELD score, and overall survival rates of the patients. However, most of these trials were performed on a small number of patients for short-term follow-up. Though this cellular-based therapy appears to be safe and tolerable especially the autologous type, still the biological behavior of these cells could not be expected in the long run regarding the toxicity, immunogenicity, and tumorigenesis that had been developed in many patients. Therefore, this stem cell-based therapy should be evaluated after long-term follow-up, taking into consideration the site and route of administration as well as the nature of transplanted cells according to the type of liver injury and the presence of other comorbidities. Finally, still further research is highly required to overcome the challenges that occur after a long-term therapy, as this will open a new avenue and rescue a great number of patients who had no options for treatment other than liver transplantation.

## Data Availability

Data supporting the findings are included in the manuscript, and any additional data are available at the corresponding author on request.

## Abbreviations

ACLF: Acute-on-chronic liver failure
AD-MSCs: Adipose-derived MSCs
AFB1: Aflatoxin B1
A1AT: Alpha-1 antitrypsin
ALP: Alkaline phosphatase
ARDS: Acute respiratory distress syndrome
BM-MNCs: Bone marrow mononuclear cells
BM-MSCs: Bone marrow-derived mesenchymal stem cells
CART: Chimeric antigen receptor T cells
CSCs: Cancer stem cells
CXCR4: C-X-C motif chemokine receptor 4
CK19: Cytokeratin 19
DCs: Dendritic cells
ECM: Extracellular matrix
EpCAM: Epithelial cell adhesion molecule
ESCs: Embryonic stem cells
ESLD: End-stage liver disease
GGT: Gamma-glutamyl transferase
G-CSF: Human granulocyte colony-stimulating factor.
GVHD: Graft-versus-host disease
HBV: Chronic hepatitis B virus
HCC: Hepatocellular carcinoma
HCV: Hepatitis C virus
HLCs: Hepatocyte-like cells
HSCs: Hemopoietic stem cells
HepaStem: Human MSCs
HGF: Hepatocyte growth factor
IDO: Indoleamine 2,3‐dioxygenase
IL-10: Interleukin-10
iPSCs: Induced pluripotent stem cells
ICAM-1: Intercellular adhesion molecule-1
LCSCs: Liver cancer stem cells
MELD: Model for end-stage liver disease
MHC-1: Major histocompatibility complex-1
MS: Multiple sclerosis
MSCs: Mesenchymal stem cells
NAFLD: Non-alcoholic fatty liver disease
PDGFB: Platelet-derived growth factor subunit B
PGE2: Prostaglandin 2
PBC: Primary biliary cirrhosis
PSCs: Pluripotent stem cells
SGOT: Serum glutamic oxaloacetic transaminase
SGPT: Serum glutamic pyruvic transaminase
SPIO@AuNPs: Superparamagnetic iron oxide-coated gold nanoparticles
SPCs: Stem-progenitor cells
TACE: Trans-arterial chemoembolization
TFs: Transcription factors
TGF‐β3: Transforming growth factor-β3
TGF-b1: Transforming growth factor beta-1
UC-MSCs: Umbilical cord-derived MSCs
VEGF: Vascular endothelial growth factor
WHO: World Health Organization

## References

[1] Sung H, Ferlay J, Siegel RL, et al. Global Cancer Statistics 2020: GLOBOCAN estimates of incidence and mortality worldwide for 36 cancers in 185 countries. CA Cancer J Clin. 2021;71(3):209–49. 10.3322/caac.21660. 33538338 10.3322/caac.21660

[2] Siegel RL, Miller KD, Jemal A. Cancer statistics, 2019. CA Cancer J Clin. 2019;69(1):7–34. 10.3322/caac.21551. 30620402 10.3322/caac.21551

[3] Howell J, Pedrana A, Schroeder SE, et al. A global investment framework for the elimination of hepatitis b. J Hepatol. 2021;74(3):535–49. 10.1016/j.jhep.2020.09.013. 32971137 10.1016/j.jhep.2020.09.013 PMC7505744

[4] Schweitzer A, Horn J, Mikolajczyk RT, Krause G, Ott JJ. Estimations of worldwide prevalence of chronic hepatitis B virus infection: a systematic review of data published between 1965 and 2013. Lancet. 2015;386(10003):1546–55. 10.1016/S0140-6736(15)61412-X. 26231459 10.1016/S0140-6736(15)61412-X

[5] Wild CP, Miller JD, Groopman JD, editors. Mycotoxin control in low- and middle-income countries. International Agency for Research on Cancer: Lyon; 2015. PMID: 27030861. 27030861

[6] Cheng P, Cheng Y, Su MX, et al. Bicluster and pathway enrichment analysis of HCV-induced cirrhosis and hepatocellular carcinoma. Asian Pac J Cancer Prev. 2012;13(8):3741–5. 10.7314/apjcp.2012.13.8.3741. 23098464 10.7314/apjcp.2012.13.8.3741

[7] Younossi ZM, Blissett D, Blissett R, et al. The economic and clinical burden of nonalcoholic fatty liver disease in the United States and Europe. Hepatology. 2016;64(5):1577–86. 10.1002/hep.28785. 27543837 10.1002/hep.28785

[8] Lu W, Jin XL, Yang C, et al. Comparison of efficacy between TACE combined with apatinib and TACE alone in the treatment of intermediate and advanced hepatocellular carcinoma: a single-center randomized controlled trial. Cancer Biol Ther. 2017;18(6):433–8. 10.1080/15384047.2017.1323589. 28548587 10.1080/15384047.2017.1323589 PMC5536939

[9] Lee TK, Guan XY, Ma S. Cancer stem cells in hepatocellular carcinoma - from origin to clinical implications. Nat Rev Gastroenterol Hepatol. 2022;19(1):26–44. 10.1038/s41575-021-00508-3. 34504325 10.1038/s41575-021-00508-3

[10] Huang A, Yang XR, Chung WY, Dennison AR, Zhou J. Targeted therapy for hepatocellular carcinoma. Signal Transduct Target Ther. 2020;5(1):146. 10.1038/s41392-020-00264-x. 32782275 10.1038/s41392-020-00264-x PMC7419547

[11] Anwanwan D, Singh SK, Singh S, Saikam V, Singh R. Challenges in liver cancer and possible treatment approaches. Biochim Biophys Acta Rev Cancer. 2020;1873(1):188314. 10.1016/j.bbcan.2019.188314. 31682895 10.1016/j.bbcan.2019.188314 PMC6981221

[12] Chen Z, Xie H, Hu M, et al. Recent progress in treatment of hepatocellular carcinoma. Am J Cancer Res. 2020;10(9):2993–3036. PMID: 33042631. 33042631 PMC7539784

[13] Jadlowiec CC, Taner T. Liver transplantation: current status and challenges. World J Gastroenterol. 2016;22:4438–45. 10.3748/wjg.v22.i18.4438. 27182155 10.3748/wjg.v22.i18.4438 PMC4858627

[14] Zakrzewski W, Dobrzyński M, Szymonowicz M, Rybak Z. Stem cells: past, present, and future. Stem Cell Res Ther. 2019;10(1):68. 10.1186/s13287-019-1165-5. 30808416 10.1186/s13287-019-1165-5 PMC6390367

[15] Sobhani A, Khanlarkhani N, Baazm M, et al. Multipotent stem cell and current application. Acta Med Iran. 2017;55(1):6–23. PMID: 28188938. 28188938

[16] Gargett CE. Adult stem cells in the human endometrium. In: Simon C, Pellicer A, editors. Stem Cells in Human Reproduction: Basic Science and Therapeutic Potential. 2nd ed. London: Informa Healthcare; 2009. p. 160–76.

[17] Larijani B, Esfahani EN, Amini P, et al. Stem cell therapy in treatment of different diseases. Acta Med Iran. 2012;50(2):79–96. PMID: 22359076. 22359076

[18] Jaenisch R, Young R. Stem cells, the molecular circuitry of pluripotency and nuclear reprogramming. Cell. 2008;132:567–82. 10.1016/j.cell.2008.01.015. 18295576 10.1016/j.cell.2008.01.015 PMC4142810

[19] Li L, Xie T. Stem cell niche: structure and function. Ann Rev Cell Dev Biol. 2005;21:605–31. 10.1146/annurev.cellbio.21.012704.131525. 16212509 10.1146/annurev.cellbio.21.012704.131525

[20] Fu RH, Wang YC, Liu SP, Huang CM, Kang YH, Tsai CH, et al. Differentiation of stem cells: strategies for modifying surface biomaterials. Cell Transplant. 2010;20:37–47. 10.3727/096368910X532756. 21054953 10.3727/096368910X532756

[21] Bibber B, Sinha G, Lobba AR, Greco SJ, Rameshwar P. A review of stem cell translation and potential confounds by cancer stem cells. Stem Cells Int. 2013;2013:241048. 10.1155/2013/241048. 24385986 10.1155/2013/241048 PMC3872439

[22] Takahashi K, Yamanaka S. Induction of pluripotent stem cells from mouse embryonic and adult fibroblast cultures by defined factors. Cell. 2006;126(4):663–76. 10.1016/j.cell.2006.07.024. 16904174 10.1016/j.cell.2006.07.024

[23] Niibe K, Kawamura Y, Araki D, et al. Purified mesenchymal stem cells are an efficient source for iPS cell induction. PLoS One. 2011;6:e17610. 10.1371/journal.pone.0017610. 21412425 10.1371/journal.pone.0017610 PMC3055883

[24] Liu J. Induced pluripotent stem cell-derived neural stem cells: new hope for stroke? Stem Cell Res Ther. 2013;4:115. 10.1186/scrt326. 24067059 10.1186/scrt326 PMC3854776

[25] Sun S, Li Z, Glencer P, et al. Bringing the age-related macular degeneration high-risk allele age-related maculopathy susceptibility 2 into focus with stem cell technology. Stem Cell Res Ther. 2017;8(1):135. 10.1186/s13287-017-0584-4. 28583181 10.1186/s13287-017-0584-4 PMC5460466

[26] Zhao D, Cui D, Wang B, et al. Treatment of early-stage osteonecrosis of the femoral head with autologous implantation of bone marrow-derived and cultured mesenchymal stem cells. Bone. 2012;50(1):325–30. 10.1016/j.bone.2011.11.002. 22094904 10.1016/j.bone.2011.11.002

[27] Shahjalal HM, Dayem AA, Lim KM, Jeon TI, Cho SG. Generation of pancreatic β cells for treatment of diabetes: advances and challenges. Stem Cell ResTher. 2018;9:355. 10.1186/s13287-018-1099-3. 10.1186/s13287-018-1099-3 PMC6310974 30594258

[28] Smith CL, Chaichana KL, Lee YM, et al. Pre-exposure of human adipose mesenchymal stem cells to soluble factors enhances their homing to brain cancer. Stem Cells Transl Med. 2015;4(3):239–51. 10.5966/sctm.2014-0149. 25646527 10.5966/sctm.2014-0149 PMC4339851

[29] Ma F, Chen D, Chen F, et al. Human umbilical cord mesenchymal stem cells promote breast cancer metastasis by interleukin-8- and interleukin-6-dependent induction of CD44(+)/CD24(-) cells. Cell Transplant. 2015;24(12):2585–99. 10.3727/096368915X687462. 25695620 10.3727/096368915X687462

[30] Xie C, Yang Z, Suo Y. Systemically infused mesenchymal stem cells show different homing profiles in healthy and tumor mouse models. Stem Cells Transl Med. 2017;6(4):1120–31. 10.1002/sctm.16-0204. 28205428 10.1002/sctm.16-0204 PMC5442841

[31] Yamashita T, Wang XW. Cancer stem cells in the development of liver cancer. J Clin Invest. 2013;123(5):1911–8. 10.1172/JCI66024. 23635789 10.1172/JCI66024 PMC3635728

[32] Ye J, Sun D, Yu Y, Yu J. Osthole resensitizes CD133+ hepatocellular carcinoma cells to cisplatin treatment via PTEN/AKT pathway. Aging (Albany NY). 2020;12(14):14406–17. 10.18632/aging.103484. 32673286 10.18632/aging.103484 PMC7425450

[33] Wang X, Wang R, Bai S, et al. Musashi2 contributes to the maintenance of CD44v6+ liver cancer stem cells via notch1 signaling pathway. J Exp Clin Cancer Res. 2019;38(1):505. 10.1186/s13046-019-1508-1. Published 2019 Dec 30. 31888685 10.1186/s13046-019-1508-1 PMC6936093

[34] Zheng H, Pomyen Y, Hernandez MO, et al. Single-cell analysis reveals cancer stem cell heterogeneity in hepatocellular carcinoma. Hepatology. 2018;68(1):127–40. 10.1002/hep.29778. 29315726 10.1002/hep.29778 PMC6033650

[35] Chen L, Wu M, Ji C, et al. Silencing transcription factor FOXM1 represses proliferation, migration, and invasion while inducing apoptosis of liver cancer stem cells by regulating the expression of ALDH2. IUBMB Life. 2020;72(2):285–95. 10.1002/iub.2166. 31580537 10.1002/iub.2166

[36] Liu L, Borlak J. Advances in liver cancer stem cell isolation and their characterization. Stem Cell Rev Rep. 2021;17(4):1215–38. 10.1007/s12015-020-10114-6. 33432485 10.1007/s12015-020-10114-6

[37] Zeng C, Zhang Y, Park SC, et al. CD34(+) Liver cancer stem cells were formed by fusion of hepatobiliary stem/progenitor cells with hematopoietic precursor-derived myeloid intermediates. Stem Cells Dev. 2015;24(21):2467–78. 10.1089/scd.2015.0202. 26192559 10.1089/scd.2015.0202 PMC4620524

[38] Song Y, Park IS, Kim J, Seo HR. Actinomycin D inhibits the expression of the cystine/glutamate transporter xCT via attenuation of CD133 synthesis in CD133+ HCC. Chem Biol Interact. 2019;309:108713. 10.1016/j.cbi.2019.06.026. 31226288 10.1016/j.cbi.2019.06.026

[39] Zhang K, Che S, Su Z, et al. CD90 promotes cell migration, viability and sphere forming ability of hepatocellular carcinoma cells. Int J Mol Med. 2018;41(2):946–54. 10.3892/ijmm.2017.3314. 29251325 10.3892/ijmm.2017.3314 PMC5752240

[40] Rozeik MS, Hammam OA, Ali AI, et al. Evaluation of CD44 and CD133 as markers of liver cancer stem cells in Egyptian patients with HCV-induced chronic liver diseases versus hepatocellular carcinoma. Electron Physician. 2017;9(7):4708–17. 10.19082/4708. 28894525 10.19082/4708 PMC5586983

[41] Yang XR, Xu Y, Yu B, et al. High expression levels of putative hepatic stem/progenitor cell biomarkers related to tumour angiogenesis and poor prognosis of hepatocellular carcinoma. Gut. 2010;59(7):953–62. 10.1136/gut.2008.176271. 20442200 10.1136/gut.2008.176271

[42] Zhu Z, Hao X, Yan M, et al. Cancer stem/progenitor cells are highly enriched in CD133+CD44+ population in hepatocellular carcinoma. Int J Cancer. 2010;126(9):2067–78. 10.1002/ijc.24868. 19711346 10.1002/ijc.24868

[43] Ma S, Chan KW, Lee TK, et al. Aldehyde dehydrogenase discriminates the CD133 liver cancer stem cell populations. Mol Cancer Res. 2008;6(7):1146–53. 10.1158/1541-7786.MCR-08-0035. 18644979 10.1158/1541-7786.MCR-08-0035

[44] Hu C, Li H, Li J, et al. Analysis of ABCG2 expression and side population identifies intrinsic drug efflux in the HCC cell line MHCC-97L and its modulation by Akt signaling. Carcinogenesis. 2008;29(12):2289–97. 10.1093/carcin/bgn223. 18820285 10.1093/carcin/bgn223

[45] Liu S, Li N, Yu X, et al. Expression of intercellular adhesion molecule 1 by hepatocellular carcinoma stem cells and circulating tumor cells. Gastroenterology. 2013;144(5):1031-1041.e10. 10.1053/j.gastro.2013.01.046. 23376424 10.1053/j.gastro.2013.01.046

[46] Wang L, Li Y, Xu J, et al. Quantified postsurgical small cell size CTCs and EpCAM+ circulating tumor stem cells with cytogenetic abnormalities in hepatocellular carcinoma patients determine cancer relapse. Cancer Lett. 2018;412:99–107. 10.1016/j.canlet.2017.10.004. 29031565 10.1016/j.canlet.2017.10.004

[47] Sun YF, Xu Y, Yang XR, et al. Circulating stem cell-like epithelial cell adhesion molecule-positive tumor cells indicate poor prognosis of hepatocellular carcinoma after curative resection. Hepatology. 2013;57(4):1458–68. 10.1002/hep.26151. 23175471 10.1002/hep.26151

[48] Guo W, Sun YF, Shen MN, et al. Circulating tumor cells with stem-like phenotypes for diagnosis, prognosis, and therapeutic response evaluation in hepatocellular carcinoma. Clin Cancer Res. 2018;24(9):2203–13. 10.1158/1078-0432.CCR-17-1753. 29374055 10.1158/1078-0432.CCR-17-1753

[49] Liu YC, Yeh CT, Lin KH. Cancer stem cell functions in hepatocellular carcinoma and comprehensive therapeutic strategies. Cells. 2020;9(6):1331. 10.3390/cells9061331. Published 2020 May 26. 32466488 10.3390/cells9061331 PMC7349579

[50] Chan KK, Lo RC. Deregulation of frizzled receptors in hepatocellular carcinoma. Int J Mol Sci. 2018;19(1):313. 10.3390/ijms19010313. 29361730 10.3390/ijms19010313 PMC5796257

[51] Le PN, McDermott JD, Jimeno A. Targeting the Wnt pathway in human cancers: therapeutic targeting with a focus on OMP-54F28. Pharmacol Ther. 2015;146:1–11. 10.1016/j.pharmthera.2014.08.005. 25172549 10.1016/j.pharmthera.2014.08.005 PMC4304994

[52] Wu CX, Xu A, Zhang CC, et al. Notch inhibitor PF-03084014 inhibits hepatocellular carcinoma growth and metastasis via suppression of cancer stemness due to reduced activation of Notch1-Stat3. Mol Cancer Ther. 2017;16(8):1531–43. 10.1158/1535-7163.MCT-17-0001. 28522590 10.1158/1535-7163.MCT-17-0001

[53] Serova M, Tijeras-Raballand A, Dos Santos C, et al. Effects of TGF-beta signalling inhibition with galunisertib (LY2157299) in hepatocellular carcinoma models and in ex vivo whole tumor tissue samples from patients. Oncotarget. 2015;6(25):21614–27. 10.18632/oncotarget.4308. 26057634 10.18632/oncotarget.4308 PMC4673290

[54] Ji S, Ma Y, Xing X, et al. Suppression of CD13 enhances the cytotoxic effect of chemotherapeutic drugs in hepatocellular carcinoma cells. Front Pharmacol. 2021;12:660377. 10.3389/fphar.2021.660377. 34045966 10.3389/fphar.2021.660377 PMC8144446

[55] Ogawa K, Tanaka S, Matsumura S, et al. EpCAM-targeted therapy for human hepatocellular carcinoma. Ann Surg Oncol. 2014;21(4):1314–22. 10.1245/s10434-013-3430-7. 24370904 10.1245/s10434-013-3430-7

[56] Sanaei M, Kavoosi F. Effect of zebularine on apoptotic pathways in hepatocellular carcinoma cell lines. Int J Prev Med. 2023;14:63. 10.4103/ijpvm.ijpvm_191_21. 37351028 10.4103/ijpvm.ijpvm_191_21 PMC10284256

[57] Zeng SS, Yamashita T, Kondo M, et al. The transcription factor SALL4 regulates stemness of EpCAM-positive hepatocellular carcinoma. J Hepatol. 2014;60(1):127–34. 10.1016/j.jhep.2013.08.024. 24012616 10.1016/j.jhep.2013.08.024

[58] Sun JC, Pan K, Chen MS, et al. Dendritic cells-mediated CTLs targeting hepatocellular carcinoma stem cells. Cancer Biol Ther. 2010;10(4):368–75. 10.4161/cbt.10.4.12440. 20581468 10.4161/cbt.10.4.12440

[59] Choi YJ, Park SJ, Park YS, et al. EpCAM peptide-primed dendritic cell vaccination confers significant anti-tumor immunity in hepatocellular carcinoma cells. PLoS One. 2018;13(1):e0190638. 10.1371/journal.pone.0190638. 29298343 10.1371/journal.pone.0190638 PMC5752035

[60] Wang Y, Chen M, Wu Z, et al. CD133-directed CAR T cells for advanced metastasis malignancies: a phase I trial. Oncoimmunology. 2018;7(7):e1440169. 10.1080/2162402X.2018.1440169. 29900044 10.1080/2162402X.2018.1440169 PMC5993480

[61] Midha S, Jain KG, Bhaskar N, et al. Tissue-specifc mesenchymal stem cell-dependent osteogenesis in highly porous chitosan-based bone analogs. Stem Cells Transl Med. 2020. 10.1002/sctm.19-0385. 10.1002/sctm.19-0385 PMC7848378 33049125

[62] Vaananen HK. Mesenchymal stem cells. Ann Med. 2005;37(7):469–79. 10.1080/07853890500371957. 16278160 10.1080/07853890500371957

[63] Mushahary D, Spittler A, Kasper C, Weber V, Charwat V. Isolation, cultivation, and characterization of human mesenchymal stem cells. Cytometry A. 2018;93(1):19–31. 10.1002/cyto.a.23242. 29072818 10.1002/cyto.a.23242

[64] Martínez-Carrasco R, Sánchez-Abarca LI, Nieto-Gómez C, et al. Subconjunctival injection of mesenchymal stromal cells protects the cornea in an experimental model of GVHD. Ocul Surf. 2019;17(2):285–94. 10.1016/j.jtos.2019.01.001. 30630121 10.1016/j.jtos.2019.01.001

[65] Petrou P, Gothelf Y, Argov Z, et al. Safety and clinical effects of mesenchymal stem cells secreting neurotrophic factor transplantation in patients with amyotrophic lateral sclerosis: results of phase 1/2 and 2a clinical trials. JAMA Neurol. 2016;73(3):337–44. 10.1001/jamaneurol.2015.4321. 26751635 10.1001/jamaneurol.2015.4321

[66] Zhao K, Liu Q. The clinical application of mesenchymal stromal cells in hematopoietic stem cell transplantation. J Hematol Oncol. 2016;9(1):46. 10.1186/s13045-016-0276-z. 27193054 10.1186/s13045-016-0276-z PMC4870746

[67] Park YB, Ha CW, Lee CH, Yoon YC, Park YG. Cartilage regeneration in osteoarthritic patients by a composite of allogeneic umbilical cord blood-derived mesenchymal stem cells and hyaluronate hydrogel: results from a clinical trial for safety and proof-of-concept with 7 years of extended follow-up. Stem Cells Transl Med. 2017;6(2):613–21. 10.5966/sctm.2016-0157. 28191757 10.5966/sctm.2016-0157 PMC5442809

[68] Rubin R. Unproven but proftable: the boom in US stem cell clinics. JAMA. 2018;320(14):1421–3. 10.1001/jama.2018.13861. 30326510 10.1001/jama.2018.13861

[69] Wang L, Li J, Liu H, et al. Pilot study of umbilical cord-derived mesenchymal stem cell transfusion in patients with primary biliary cirrhosis. J Gastroenterol Hepatol. 2013;28(Suppl 1):85–92. 10.1111/jgh.12029. 23855301 10.1111/jgh.12029

[70] Zhang YC, Liu W, Fu BS, et al. Therapeutic potentials of umbilical cord-derived mesenchymal stromal cells for ischemic-type biliary lesions following liver transplantation. Cytotherapy. 2017;19(2):194–9. 10.1016/j.jcyt.2016.11.005. 27964826 10.1016/j.jcyt.2016.11.005

[71] Xu WX, He HL, Pan SW, et al. Combination treatments of plasma exchange and umbilical cord-derived mesenchymal stem cell transplantation for patients with hepatitis B virus-related acute-on-chronic liver failure: a clinical trial in China. Stem Cells Int. 2019;2019:4130757. 30863450 10.1155/2019/4130757 PMC6378797

[72] Yu SJ, Chen LM, Lyu S, et al. ZhonghuaGan Zang Bing Za Zhi. 2016;24(1):51-55. 10.3760/cma.j.issn.1007-3418.2016.01.010. 10.3760/cma.j.issn.1007-3418.2016.01.010 PMC12770687 26983390

[73] Jia Y, Shu X, Yang X, et al. Enhanced therapeutic effects of umbilical cord mesenchymal stem cells after prolonged treatment for HBV-related liver failure and liver cirrhosis. Stem Cell Res Ther. 2020;11(1):277. 10.1186/s13287-020-01787-4. Published 2020 Jul 10. 32650827 10.1186/s13287-020-01787-4 PMC7350639

[74] Zhang K, Sun H, Cao H, et al. The impact of recipient age on the effects of umbilical cord mesenchymal stem cells on HBV-related acute-on-chronic liver failure and liver cirrhosis. Stem Cell Res Ther. 2021;12(1):466. 10.1186/s13287-021-02544-x. Published 2021 Aug 20. 34416908 10.1186/s13287-021-02544-x PMC8379867

[75] Nevens F, Gustot T, Laterre PF, et al. A phase II study of human allogeneic liver-derived progenitor cell therapy for acute-on-chronic liver failure and acute decompensation. JHEP Rep. 2021;3(4):100291. 10.1016/j.jhepr.2021.100291. Published 2021 Apr 18. 34169246 10.1016/j.jhepr.2021.100291 PMC8207211

[76] Lin BL, Chen JF, Qiu WH, et al. Allogeneic bone marrow-derived mesenchymal stromal cells for hepatitis B virus-related acute-on-chronic liver failure: a randomized controlled trial. Hepatology. 2017;66(1):209–19. 10.1002/hep.29189. 28370357 10.1002/hep.29189

[77] Dimmeler S, Ding S, Rando TA, Trounson A. Translational strategies and challenges in regenerative medicine. Nat Med. 2014;20(8):814–21. 10.1038/nm.3627. 25100527 10.1038/nm.3627

[78] Chan JL, Tang KC, Patel AP, Bonilla LM, Pierobon N, Ponzio NM, et al. Antigen-presenting property of mesenchymal stem cells occurs during a narrow window at low levels of interferon-gamma. Blood. 2006;107(12):4817–24. 10.1182/blood-2006-01-0057. 16493000 10.1182/blood-2006-01-0057 PMC1895812

[79] Zhou T, Yuan Z, Weng J, et al. Challenges and advances in clinical applications of mesenchymal stromal cells. J Hematol Oncol. 2021;14(1):24. 10.1186/s13045-021-01037-x. 33579329 10.1186/s13045-021-01037-x PMC7880217

[80] Conrad C, Niess H, Huss R, et al. Multipotent mesenchymal stem cells acquire a lymphendothelial phenotype and enhance lymphatic regeneration in vivo. Circulation. 2009;119(2):281–9. 10.1161/CIRCULATIONAHA.108.793208. 19118255 10.1161/CIRCULATIONAHA.108.793208

[81] Haga H, Yan IK, Takahashi K, Wood J, Zubair A, Patel T. Tumour cell-derived extracellular vesicles interact with mesenchymal stem cells to modulate the microenvironment and enhance cholangiocarcinoma growth. J Extracell Vesicles. 2015;4:24900. 10.3402/jev.v4.24900. 25557794 10.3402/jev.v4.24900 PMC4283029

[82] Pankaj P, Zhang Q, Bai XL, Liang TB. Autologous bone marrow transplantation in decompensated liver: systematic review and meta-analysis. World J Gastroenterol. 2015;21(28):8697–710. 10.3748/wjg.v21.i28.8697. 26229412 10.3748/wjg.v21.i28.8697 PMC4515851

[83] Mora C, Serzanti M, Consiglio A, Memo M, Dell’Era P. Clinical potentials of human pluripotent stem cells. Cell Biol Toxicol. 2017;33(4):351–60. 10.1007/s10565-017-9384-y. 28176010 10.1007/s10565-017-9384-y

[84] Hu C, Li L. Preconditioning influences mesenchymal stem cell properties in vitro and in vivo. J Cell Mol Med. 2018;22:1428–42. 10.1111/jcmm.13492. 29392844 10.1111/jcmm.13492 PMC5824372

[85] Mohamed Y, Basyony MA, El-Desouki NI, Abdo WS, El-Magd MA. The potential therapeutic effect for melatonin and mesenchymal stem cells on hepatocellular carcinoma. Biomedicine (Taipei). 2019;9:24. 10.1051/bmdcn/2019090424. 31724939 10.1051/bmdcn/2019090424 PMC6855194

[86] Driscoll J, Patel T. The mesenchymal stem cell secretome as an acellular regenerative therapy for liver disease. J Gastroenterol. 2019;54:763–73. 10.1007/s00535-019-01599-1. 31270691 10.1007/s00535-019-01599-1 PMC6698261

[87] Bartholomew A, et al. Mesenchymal stem cells suppress lymphocyte proliferation in vitro and prolong skin graft survival in vivo. Exp Hema tol. 2002;30(1):42–8. 10.1016/S0301-472X(01)00769-X. 10.1016/s0301-472x(01)00769-x 11823036

[88] Djouad F, et al. Immunosuppressive effect of mesenchymal stem cells favours tumor growth in allogeneic animals. Blood. 2003;102(10):3837–44. 10.1182/blood-2003-04-1193. 12881305 10.1182/blood-2003-04-1193

[89] Hu C, Wu Z, Li L. Pre-treatments enhance the therapeutic effects of mesenchymal stem cells in liver diseases. J Cell Mol Med. 2020;24(1):40–9. 10.1111/jcmm.14788. 31691463 10.1111/jcmm.14788 PMC6933358

[90] Ge W, et al. Infusion of mesenchymal stem cells and rapamycin syner gize to attenuate alloimmune responses and promote cardiac allograft tolerance. Am J Transplant. 2009;9(8):1760–72. 10.1111/j.1600-6143.2010.03186.x. 19563344 10.1111/j.1600-6143.2009.02721.x

[91] Waterman RS, et al. A new mesenchymal stem cell (MSC) paradigm: polarization into a pro-infammatory MSC1 or an immunosuppressive MSC2 phenotype. PLoS One. 2010;5(4):e10088. 10.1371/journal.pone.0010088. 20436665 10.1371/journal.pone.0010088 PMC2859930

[92] Zhang Y, Zhang J, Yi H, et al. A novel MSC-based immune induction strategy for ABO-incompatible liver transplantation: a phase I/II randomized, open-label, controlled trial. Stem Cell Res Ther. 2021;12(1):244. 10.1186/s13287-021-02246-4. Published 2021 Apr 16. 33863383 10.1186/s13287-021-02246-4 PMC8050996

[93] Rattigan Y, Hsu JM, Mishra PJ, Glod J, Banerjee D. Interleukin 6 mediated recruitment of mesenchymal stem cells to the hypoxic tumor milieu. Exp Cell Res. 2010;316(20):3417–24. 10.1016/j.yexcr.2010.07.002. 20633553 10.1016/j.yexcr.2010.07.002

[94] Schär MO, Diaz-Romero J, Kohl S, Zumstein MA, Nesic D. Platelet-rich concentrates differentially release growth factors and induce cell migration in vitro. Clin Orthop Relat Res. 2015;473(5):1635–43. 10.1007/s11999-015-4192-2. 25690170 10.1007/s11999-015-4192-2 PMC4385378

[95] Kalimuthu S, Oh JM, Gangadaran P, et al. In vivo tracking of chemokine receptor CXCR4-engineered mesenchymal stem cell migration by optical molecular imaging [published correction appears in Stem Cells Int. 2020;2020:8275897]. Stem Cells Int. 2017;2017:8085637. 10.1155/2017/8085637. 10.1155/2017/8085637 PMC5505027 28740515

[96] Honczarenko M, Le Y, Swierkowski M, Ghiran I, Glodek AM, Silberstein LE. Human bone marrow stromal cells express a distinct set of biologically functional chemokine receptors. Stem Cells. 2006;24(4):1030–41. 10.1634/stemcells.2005-0319. 16253981 10.1634/stemcells.2005-0319

[97] Bao Q, Zhao Y, Niess H, et al. Mesenchymal stem cell-based tumor-targeted gene therapy in gastrointestinal cancer. Stem Cells Dev. 2012;21(13):2355–63. 10.1089/scd.2012.0060. 22530882 10.1089/scd.2012.0060 PMC3424981

[98] Zhao J, Vykoukal J, Abdelsalam M, et al. Stem cell-mediated delivery of SPIO-loaded gold nanoparticles for the theranosis of liver injury and hepatocellular carcinoma. Nanotechnology. 2014;25(40):405101. 10.1088/0957-4484/25/40/405101. 25211057 10.1088/0957-4484/25/40/405101 PMC4414337

[99] Golinelli G, Mastrolia I, Aramini B, et al. Arming mesenchymal stromal/stem cells against cancer: has the time come? Front Pharmacol. 2020;11:529921. 10.3389/fphar.2020.529921. 33117154 10.3389/fphar.2020.529921 PMC7553050

[100] Schug C, Urnauer S, Jaeckel C, et al. TGFB1-driven mesenchymal stem cell-mediated NIS gene transfer. Endocr Relat Cancer. 2019;26(1):89–101. 10.1530/ERC-18-0173. 30121623 10.1530/ERC-18-0173

[101] Peng L, Xie DY, Lin BL, et al. Autologous bone marrow mesenchymal stem cell transplantation in liver failure patients caused by hepatitis B: short-term and long-term outcomes. Hepatology. 2011;54(3):820–8. 10.1002/hep.24434. 21608000 10.1002/hep.24434

[102] Suk KT, Yoon JH, Kim MY, et al. Transplantation with autologous bone marrow-derived mesenchymal stem cells for alcoholic cirrhosis: phase 2 trial. Hepatology. 2016;64(6):2185–97. 10.1002/hep.28693. 27339398 10.1002/hep.28693

[103] Sakai Y, Takamura M, Seki A, et al. Phase I clinical study of liver regenerative therapy for cirrhosis by intrahepatic arterial infusion of freshly isolated autologous adipose tissue-derived stromal/stem (regenerative) cell. Regen Ther. 2017;6:52–64. 10.1016/j.reth.2016.12.001. Published 2017 Mar 9. 30271839 10.1016/j.reth.2016.12.001 PMC6134901

[104] Salama H, Zekri AR, Medhat E, Al Alim SA, Ahmed OS, Bahnassy AA, Lotfy MM, Ahmed R, Musa S. Peripheral vein infusion of autologous mesenchymal stem cells in Egyptian HCV-positive patients with end-stage liver disease. Stem Cell Res Ther. 2014;5(3):70. 10.1186/scrt459. 24886681 10.1186/scrt459 PMC4097846

[105] Josse C, Schoemans R, Niessen NA, et al. Systematic chromosomal aberrations found in murine bone marrow-derived mesenchymal stem cells. Stem Cells Dev. 2010;19(8):1167–73. 10.1089/scd.2009.0264. 20109032 10.1089/scd.2009.0264

[106] Røsland GV, Svendsen A, Torsvik A, et al. Long-term cultures of bone marrow-derived human mesenchymal stem cells frequently undergo spontaneous malignant transformation. Cancer Res. 2009;69(13):5331–9. 10.1158/0008-5472.CAN-08-4630. 19509230 10.1158/0008-5472.CAN-08-4630

[107] Guo L, Du J, Yuan DF, et al. Optimal H2O2 preconditioning to improve bone marrow mesenchymal stem cells’ engraftment in wound healing. Stem Cell Res Ther. 2020;11(1):434. 10.1186/s13287-020-01910-5. 33032649 10.1186/s13287-020-01910-5 PMC7545926

[108] Chawla S, Das A. Preclinical-to-clinical innovations in stem cell therapies for liver regeneration. Curr Res Transl Med. 2023;71(1):103365. 10.1016/j.retram.2022.103365. 36427419 10.1016/j.retram.2022.103365

[109] Catani L, Sollazzo D, Bianchi E, et al. Molecular and functional characterization of CD133+ stem/progenitor cells infused in patients with end-stage liver disease reveals their interplay with stromal liver cells. Cytotherapy. 2017;19(12):1447–61. 10.1016/j.jcyt.2017.08.001. 28917627 10.1016/j.jcyt.2017.08.001

[110] Spahr L, Chalandon Y, Terraz S, et al. Autologous bone marrow mononuclear cell transplantation in patients with decompensated alcoholic liver disease: a randomized controlled trial. PLoS One. 2013;8(1):e53719. 10.1371/journal.pone.0053719. 23341981 10.1371/journal.pone.0053719 PMC3544843

[111] Esmaeilzadeh A, Ommati H, Kooshyar MM, et al. Autologous bone marrow stem cell transplantation in liver cirrhosis after correcting nutritional anomalies, a controlled clinical study. Cell J. 2019;21(3):268–73. 10.22074/cellj.2019.6108. 31210432 10.22074/cellj.2019.6108 PMC6582418

[112] Lyra AC, Soares MB, da Silva LF, et al. Infusion of autologous bone marrow mononuclear cells through hepatic artery results in a short-term improvement of liver function in patients with chronic liver disease: a pilot randomized controlled study. Eur J Gastroenterol Hepatol. 2010;22(1):33–42. 10.1097/MEG.0b013e32832eb69a. 19654548 10.1097/MEG.0b013e32832eb69a

[113] Mohamadnejad M, Vosough M, Moossavi S, et al. Intraportal infusion of bone marrow mononuclear or CD133+ cells in patients with decompensated cirrhosis: a double-blind randomized controlled trial. Stem Cells Transl Med. 2016;5(1):87–94. 10.5966/sctm.2015-0004. 26659833 10.5966/sctm.2015-0004 PMC4704869

[114] Kim JK, Kim SJ, Kim Y, et al. Long-term follow-up of patients after autologous bone marrow cell infusion for decompensated liver cirrhosis. Cell Transplant. 2017;26(6):1059–66. 10.3727/096368917X694778. 28120743 10.3727/096368917X694778 PMC5657748

[115] Zekri AR, Salama H, Medhat E, et al. The impact of repeated autologous infusion of haematopoietic stem cells in patients with liver insufficiency. Stem Cell Res Ther. 2015;6(1):118. 10.1186/s13287-015-0106-1. 26062731 10.1186/s13287-015-0106-1 PMC4482193

[116] Margini C, Vukotic R, Brodosi L, Bernardi M, Andreone P. Bone marrow derived stem cells for the treatment of end-stage liver disease. World J Gastroenterol. 2014;20(27):9098–105. 10.3748/wjg.v20.i27.9098. 25083082 10.3748/wjg.v20.i27.9098 PMC4112892

[117] Nikeghbalian S, Pournasr B, Aghdami N, et al. Autologous transplantation of bone marrow-derived mononuclear and CD133(+) cells in patients with decompensated cirrhosis. Arch Iran Med. 2011;14(1):12–7. PMID: 21194255. 21194255

[118] Andreone P, Catani L, Margini C, et al. Reinfusion of highly purified CD133+ bone marrow-derived stem/progenitor cells in patients with end-stage liver disease: a phase I clinical trial. Dig Liver Dis. 2015;47(12):1059–66. 10.1016/j.dld.2015.08.018. 26427587 10.1016/j.dld.2015.08.018

[119] Chruscinski A, Juvet S, Moshkelgosha S, et al. Autologous hematopoietic stem cell transplantation for liver transplant recipients with recurrent primary sclerosing cholangitis: a pilot study. Transplantation. 2022;106(3):562–74. 10.1097/TP.0000000000003829. 34049362 10.1097/TP.0000000000003829

[120] Rana A, Ackah RL, Webb GJ, et al. No gains in long-term survival after liver transplantation over the past three decades. Ann Surg. 2019;269(1):20–7. 10.1097/SLA.0000000000002650. 29303806 10.1097/SLA.0000000000002650

[121] Pareja E, Gómez-Lechón MJ, Tolosa L. Induced pluripotent stem cells for the treatment of liver diseases: challenges and perspectives from a clinical viewpoint. Ann Transl Med. 2020;8(8):566. 10.21037/atm.2020.02.164. 32775367 10.21037/atm.2020.02.164 PMC7347783

[122] Antarianto RD, Pragiwaksana A, Septiana WL, Mazfufah NF, Mahmood A. Hepatocyte differentiation from iPSCs or MSCs in decellularized liver scaffold: cell-ECM adhesion, spatial distribution, and hepatocyte maturation profile. Organogenesis. 2022;18(1):2061263. 10.1080/15476278.2022.2061263. 35435152 10.1080/15476278.2022.2061263 PMC9037523

[123] Park S, In Hwang S, Kim J, et al. The therapeutic potential of induced hepatocyte-like cells generated by direct reprogramming on hepatic fibrosis. Stem Cell Res Ther. 2019;10(1):21. 10.1186/s13287-018-1127-3. Published 2019 Jan 11. 30635054 10.1186/s13287-018-1127-3 PMC6330392

[124] Chiang CH, Wu WW, Li HY, et al. Enhanced antioxidant capacity of dental pulp-derived iPSC-differentiated hepatocytes and liver regeneration by injectable HGF-releasing hydrogel in fulminant hepatic failure. Cell Transplant. 2015;24(3):541–59. 10.3727/096368915X686986. 25668102 10.3727/096368915X686986

[125] Luce E, Steichen C, Allouche M, et al. In vitro recovery of FIX clotting activity as a marker of highly functional hepatocytes in a hemophilia B iPSC model. Hepatology. 2022;75(4):866–80. 10.1002/hep.32211. 34687060 10.1002/hep.32211 PMC9299628

[126] Zhang S, Chen S, Li W, et al. Rescue of ATP7B function in hepatocyte-like cells from Wilson’s disease induced pluripotent stem cells using gene therapy or the chaperone drug curcumin. Hum Mol Genet. 2011;20(16):3176–87. 10.1093/hmg/ddr223. 21593220 10.1093/hmg/ddr223

[127] Omer L, Hudson EA, Zheng S, Hoying JB, Shan Y, Boyd NL. CRISPR correction of a homozygous low-density lipoprotein receptor mutation in familial hypercholesterolemia induced pluripotent stem cells. Hepatol Commun. 2017;1(9):886–98. 10.1002/hep4.1110. 29130076 10.1002/hep4.1110 PMC5677509

[128] Sa-Ngiamsuntorn K, Wongkajornsilp A, Phanthong P, et al. A robust model of natural hepatitis C infection using hepatocyte-like cells derived from human induced pluripotent stem cells as a long-term host. Virol J. 2016;13:59. 10.1186/s12985-016-0519-1. Published 2016 Apr 5. 27044429 10.1186/s12985-016-0519-1 PMC4820862

[129] Chen Y, Li Y, Wang X, et al. Amelioration of hyperbilirubinemia in Gunn rats after transplantation of human induced pluripotent stem cell-derived hepatocytes. Stem Cell Reports. 2015;5(1):22–30. 10.1016/j.stemcr.2015.04.017. 26074313 10.1016/j.stemcr.2015.04.017 PMC4618248

[130] Maetzel D, Sarkar S, Wang H, et al. Genetic and chemical correction of cholesterol accumulation and impaired autophagy in hepatic and neural cells derived from Niemann-Pick type C patient-specific iPS cells. Stem Cell Reports. 2014;2(6):866–80. 10.1016/j.stemcr.2014.03.014. Published 2014 May 15. 24936472 10.1016/j.stemcr.2014.03.014 PMC4050353

[131] Rashid ST, Corbineau S, Hannan N, et al. Modeling inherited metabolic disorders of the liver using human induced pluripotent stem cells. J Clin Invest. 2010;120(9):3127–36. 10.1172/JCI43122. 20739751 10.1172/JCI43122 PMC2929734

[132] Yusa K, Rashid ST, Strick-Marchand H, et al. Targeted gene correction of α1-antitrypsin deficiency in induced pluripotent stem cells. Nature. 2011;478(7369):391–4. 10.1038/nature10424. Published 2011 Oct 12. 21993621 10.1038/nature10424 PMC3198846

[133] Domae K, Miyagawa S, Yoshikawa Y, et al. Clinical outcomes of autologous stem cell-patch implantation for patients with heart failure with nonischemic dilated cardiomyopathy. J Am Heart Assoc. 2021;10(13):e008649. 10.1161/JAHA.117.008649. 34212772 10.1161/JAHA.117.008649 PMC8403293

[134] Takahashi J. iPS cell-based therapy for Parkinson’s disease: a Kyoto trial. Regen Ther. 2020;13:18–22. 10.1016/j.reth.2020.06.002. 33490319 10.1016/j.reth.2020.06.002 PMC7794047

[135] Kim JY, Nam Y, Rim YA, Ju JH. Review of the current trends in clinical trials involving induced pluripotent stem cells. Stem Cell Rev Rep. 2022;18(1):142–54. 10.1007/s12015-021-10262-3. 34532844 10.1007/s12015-021-10262-3 PMC8445612

[136] Tolosa L, Pareja E, Gómez-Lechón MJ. Clinical application of pluripotent stem cells: an alternative cell-based therapy for treating liver diseases? Transplantation. 2016;100(12):2548–57. 10.1097/TP.0000000000001426. 27495745 10.1097/TP.0000000000001426

